# Supplementary material for: Cardiac Events Potentially Associated to Remdesivir: An Analysis from the European Spontaneous Adverse Event Reporting System
Source: Pharmaceuticals (Basel). 2021 Jun 25;14(7):611. doi: 10.3390/ph14070611 (PMC8308754; doi:10.3390/ph14070611)
Supplement: Supplementary file 1 [file pharmaceuticals-14-00611-s001.zip › pharmaceuticals-1246643-supplementary.pdf]

Article

Cardiac Events Potentially Associated to Remdesivir: An Analysis from the European Spontaneous Adverse Event Reporting System

Concetta Rafaniello [1,*,†], Carmen Ferrajolo [1,†], Maria Giuseppa Sullo [1], Mario Gaio [1], Alessia Zinzi [1], Cristina Scavone [1], Francesca Gargano [2], Enrico Coscioni [3], Francesco Rossi [1,‡] and Annalisa Capuano [1,‡]

[1] Campania Regional Centre for Pharmacovigilance and Pharmacoepidemiology—Department of Experimental Medicine—Section of Pharmacology "L. Donatelli", University of Campania "Luigi Vanvitelli", Via Costantinopoli, 16, 80138 Naples, Italy; carmen.ferrajolo@unicampania.it (C.F.); pina.sullo@libero.it (M.G.S.); mario.gaio@unicampania.it (M.G.); alessia.zinzi@unicampania.it (A.Z.); cristina.scavone@unicampania.it (C.S.); francesco.rossi@unicampania.it (F.R.); annalisa.capuano@unicampania.it (A.C.)
[2] Department of Anesthesia and Resuscitation, Biomedical Campus University of Rome, 00128 Rome, Italy; f.gargano@unicampus.it
[3] AGENAS—Agenzia Nazionale per i Servizi Sanitari Regionali, via Piemonte 60, 00187 Roma, Italy; coscionienrico@gmail.com
* Correspondence: concetta.rafaniello@unicampania.it; Tel. +39-81-5665805
† These authors contributed equally to this work.
‡ These authors are both lead authors.

Supplementary Table S1: Description of type of cardiac events by preferred terms MedDRA.

Peferred Term (MedDRA)	Number of events
Bradycardia	69
Cardiac arrest	52
Atrial fibrillation	20
Pulseless electrical activity	18
Sinus bradycardia	16
Acute myocardial infarction	13
Cardio-respiratory arrest	13
Tachycardia	9
Electrocardiogram QT prolonged	6
Ventricular tachycardia	6
Blood pressure decreased	5
Heart rate increased	4
Supraventricular tachycardia	4
Cardiac failure	4
Arrhythmia supraventricular	3
Arrhythmia	3
Heart rate decreased	3
Ventricular fibrillation	3
Electrocardiogram QRS complex prolonged	3
Atrial flutter	2
Pulse absent	2
Electrocardiogram ST-T change	2
Cardiovascular disorder	2

Myocardial infarction	2
Cardiogenic shock	2
Cardiopulmonary failure	2
Ventricular extrasystoles	2
Heart rate irregular	1
Electrocardiogram ST segment elevation	1
Blood pressure systolic decreased	1
Atrioventricular block complete	1
Bradyarrhythmia	1
Bundle branch block right	1
Sinus node dysfunction	1
Myocarditis	1
Sinus tachycardia	1
Supraventricular extrasystoles	1
Tachyarrhythmia	1
Tachycardia foetal	1
Cardiac disorder	1
Cardiac dysfunction	1
Myocardial ischaemia	1
Acute right ventricular failure	1
Cardiomyopathy	1
Sinoatrial block	1
